# Providing health care in rural and remote areas: lessons from the international space station

**DOI:** 10.2471/BLT.15.162628

**Published:** 2015-12-02

**Authors:** Alfred Papali

**Affiliations:** a Division of Pulmonary and Critical Care Medicine, University of Maryland School of Medicine, 110 South Paca Street, 2nd Floor, Baltimore, Maryland, 21201, United States of America.

Circumnavigating the globe every 90 minutes, 400 km above the Earth’s surface and at a speed of 27 600 km per hour, the international space station typically does not evoke thoughts of rural Haiti. This aerospace behemoth contains some of the most expensive, most advanced technology ever designed. An isolated extraterrestrial outpost of humanity, it represents a marvel of human engineering and ingenuity. It is this very isolation, ironically, that gives it something in common with rural areas in low- and middle-income countries here on Earth. In many parts of the world, basic emergency and acute medical care is lacking.^1^ Comparing the international space station’s systems with efforts underway to address the lack of rural and remote health-care services may help clinicians, researchers and policy-makers develop new ideas and improve on existing practices.

What happens when an astronaut on the space station has a medical emergency? Certainly, the entire space station cannot gently glide its way down to Earth. At least one Soyuz spacecraft is usually docked for evacuation but there are usually only a few astronauts in the space station at a time. It is not practical for several astronauts to return to Earth accompanying the patient. It would take up to 24 hours for an astronaut to return to the ground to receive medical care – precious time lost for someone in a critical condition.^2^ The United States’ National Aeronautics and Space Administration (NASA) has mitigated the risk of medical emergencies aboard the space station by training the crew medical officer and by using on-board ultrasound and Earth-based telemedicine consultation.^3^ Space flight, though, presents several challenges, such as engineering and space constraints, limited bandwidth for data transmission, a lack of advanced diagnostic equipment and the absence of a physician. How space station crew members overcome these challenges may also be applicable to rural and remote settings on Earth.

The crew medical officer – when not medically-qualified – receives approximately 60 hours of preflight training, roughly akin to the level of a paramedic in the United States of America.^2^ This basic medical training provides the practical skills needed in the event of a medical emergency. In this context, task-shifting is done in the same way that other cadres of health-care workers are trained to provide medical care traditionally provided by doctors. In terrestrial settings, task-shifting has dramatically expanded human resources in areas with a chronic shortage of health-care workers. The World Health Organization has cited task-shifting in sub-Saharan Africa as key to ensuring cost-effective access to antiretroviral medications.^4^ The benefits of this public health approach have not been limited to human immunodeficiency virus treatment, nor are they unique to low-income countries.^5^ For example, the United States has witnessed rapid growth in the number of nurse practitioners and physician assistants over the entire spectrum of medical specialties. Like the space station’s medical officer, many of these paraprofessionals function as emergency care providers in areas where doctors are scarce.

Given the size of the space station and the lack of other advanced diagnostic equipment on board, point-of-care ultrasound is essential for diagnosing the cause of medical emergencies.^6^ Studies done on the space station have shown that ultrasound imaging done by crew members who have had basic preflight training and who receive in-orbit guidance from a ground-based flight surgeon is feasible and clinically useful.^7^ NASA-funded studies in remote locations around the world have had a direct impact on the development of the space station’s ultrasound programme.^8^ On Earth, enhanced point-of-care ultrasound training is improving the diagnostic capacity of emergency care providers in remote areas. Ultrasound has been used in a wide range of situations and geographical settings including disaster zones^9^ and high-altitude locations.^10^ Better access to portable ultrasound machines coupled with task-shifting may have the potential to improve emergency and acute care provision in more settings on Earth.

Telemedicine – the provision or oversight of medical care remotely using audiovisual technology – can combine task-shifting and ultrasound access to improve the management of patients in remote settings. Aboard the space station, telemedicine is used extensively. Vital medical data and ultrasound images are routinely transmitted to ground-based flight surgeons for diagnostic and training purposes. However, data transmission is not continuous and, as in the developing world, the connection can be very slow or completely absent.^2^ Judicious use of limited technological resources is necessary in any location. Just-in-time educational modules have enabled crew members to perform complex ultrasound examinations despite the time lag in communications between the space station and the ground.^7^ These modules could be adapted to terrestrial environments with limited connectivity. In addition, NASA has tested virtual remote guidance (i.e. recorded instructional videos for use by crew members using wearable technology) as a means of overcoming connectivity barriers;^11^ this technique will soon be used in Haiti to study remote guidance of endotracheal intubation (M Walsh, personal communication, October 2015).

Although innovative ways of using technological and human resources can improve emergency care, there are limitations. Task-shifting requires people, training and cooperation between all levels of the health system. Costs, training needs, theft and unreliable power sources limit ubiquitous use of point-of-care ultrasound. Cross-border telemedicine services could improve access to medical care in remote areas but legal, cultural and sustainability issues need to be resolved.^12^ Remote telemedicine systems require the willing participation of innumerable individuals, institutions, medical professional societies, nongovernmental organizations and governments. The space station’s emergency medical contingency plans were developed methodically by national space agencies using an evidence-based, data-driven approach. This organized and purposeful approach increases the likelihood of a positive clinical outcome should a life-threatening emergency occur in space. The great challenge for emergency care on Earth will be whether such services can be developed across diverse and fragmented health systems in a coordinated fashion to optimize health outcomes, reduce costs and minimize duplication.^1^

People living in remote terrestrial villages with limited medical resources have to survive in difficult environments, and accidents and emergencies happen to people in every location on Earth. Just as in space, innovative approaches must be used to overcome the challenges of treating these emergencies wherever they occur. When thinking about the vexing barriers to improving emergency care, we can look to the sky to find solutions – the space station provides three examples of what might work. No single solution will fix the problem of providing emergency care everywhere, but task-shifting, point-of-care ultrasound and telemedicine services, if scaled up as part of an organized, collaborative approach among diverse interests, are three methods that might improve access to – and quality of – care in rural and remote areas.

## References

[1] Hirshon JM, Risko N, Calvello EJ, Stewart de Ramirez S, Narayan M, Theodosis C, et al.; Acute Care Research Collaborative at the University of Maryland Global Health Initiative. Health systems and services: the role of acute care. Bull World Health Organ. 2013 5 1;91(5):386–8. 10.2471/BLT.12.112664 23678202 PMC3646345

[2] Barratt MR, Pool SL, editors. Principles of clinical medicine for space flight. New York: Springer; 2008 10.1007/978-0-387-68164-1

[3] International Space Station medical monitoring [Internet]. Washington: National Aeronautics and Space Administration; 2015. Available from: http://www.nasa.gov/mission_pages/station/research/experiments/1025.html [cited 2015 Mar 13].

[4] Task shifting: rational redistribution of tasks among health workforce teams: global recommendations and guidelines. Geneva: World Health Organization; 2008. Available from: http://www.who.int/healthsystems/TTR-TaskShifting.pdf?ua=1 [cited 2015 Nov 27].

[5] Fulton BD, Scheffler RM, Sparkes SP, Auh EY, Vujicic M, Soucat A. Health workforce skill mix and task shifting in low income countries: a review of recent evidence. Hum Resour Health. 2011;9(1):1. 10.1186/1478-4491-9-1 21223546 PMC3027093

[6] Wagner MS, Garcia K, Martin DS. Point-of-care ultrasound in aerospace medicine: known and potential applications. Aviat Space Environ Med. 2014 7;85(7):730–9. 10.3357/ASEM.3754.2014 25022161

[7] Foale CM, Kaleri AY, Sargsyan AE, Hamilton DR, Melton S, Martin D, et al. Diagnostic instrumentation aboard ISS: just-in-time training for non-physician crewmembers. Aviat Space Environ Med. 2005 6;76(6):594–8. 15945407

[8] Cone SW, Carucci LR, Yu J, Rafiq A, Doarn CR, Merrell RC. Acquisition and evaluation of radiography images by digital camera. Telemed J E Health. 2005 4;11(2):130–6. 10.1089/tmj.2005.11.130 15857253

[9] Shorter M, Macias DJ. Portable handheld ultrasound in austere environments: use in the Haiti disaster. Prehosp Disaster Med. 2012 4;27(2):172–7. 10.1017/S1049023X12000611 22595772

[10] Otto C, Hamilton DR, Levine BD, Hare C, Sargsyan AE, Altshuler P, et al. Into thin air: extreme ultrasound on Mt Everest. Wilderness Environ Med. 2009 Fall;20(3):283–9. 10.1580/08-WEME-BR-228R2.1 19737030

[11] Martin DS, Caine TL, Matz T, Lee SMC, Stenger MB, Sargsyan AE, et al. Virtual guidance as a tool to obtain diagnostic ultrasound for spaceflight and remote environments. Aviat Space Environ Med. 2012 10;83(10):995–1000. 10.3357/ASEM.3279.2012 23066623

[12] Saliba V, Legido-Quigley H, Hallik R, Aaviksoo A, Car J, McKee M. Telemedicine across borders: a systematic review of factors that hinder or support implementation. Int J Med Inform. 2012 12;81(12):793–809. 10.1016/j.ijmedinf.2012.08.003 22975018

